# Impact of electron–phonon coupling on electron transport through T-shaped arrangements of quantum dots in the Kondo regime

**DOI:** 10.3762/bjnano.12.89

**Published:** 2021-11-12

**Authors:** Patryk Florków, Stanisław Lipiński

**Affiliations:** 1 Department of Theory of Nanostructures, Institute of Molecular Physics, Polish Academy of Sciences, M. Smoluchowskiego 17, 60-179 Poznań, Poland

**Keywords:** Fano effect, Kondo effect, polarons, quantum dots

## Abstract

We calculate the conductance through strongly correlated T-shaped molecular or quantum dot systems under the influence of phonons. The system is modelled by the extended Anderson–Holstein Hamiltonian. The finite-U mean-field slave boson approach is used to study many-body effects. Phonons influence both interference and correlations. Depending on the dot unperturbed energy and the strength of electron–phonon interaction, the system is occupied by a different number of electrons that effectively interact with each other repulsively or attractively. This leads, together with the interference effects, to different spin or charge Fano–Kondo effects.

## Introduction

As the dimension of a mesoscopic system decreases, interactions between electrons become more important and many-body resonances build up. As a consequence, new transport paths are opened. The key phenomenon of strong correlations is the Kondo effect, which arises from the coherent superposition of cotunneling processes. The latter lead to effective spin flips, in consequence of which the bound singlet state of the dot spin with the electrons of the leads is formed. This resonance is characterized by SU(2) symmetry. In nanoscopic systems SU(2) Kondo effects have been observed in semiconductor-based quantum dots (QDs) [1–4], in carbon nanotubes [5], and in molecular nanostructures [6–9]. Besides the spin, also other degrees of freedom, for example, orbital [10] or charge [11–12] can give rise to Kondo correlations. For systems with higher degeneracy, for example, in the case of fourfold spin–orbital degeneracy not only spin, but also orbital pseudo-spin can be screened. Such SU(4) Kondo effect resonances have been observed in vertical QDs [10], in capacitively coupled dots [13], and in carbon nanotubes [14–17].

There is currently also a great interest in the interplay of strong correlations and interference in multiply connected geometries, for example, in T-shaped systems, where a dot or a molecule are side-coupled to a quantum wire [18–26]. Sidewall chemical functionalization of molecular wires is already a well-established branch of research. The attached objects act as scatterers for electron transmission through the quantum wire and allow one to tune its transport properties. In T-shaped systems, the interference of different conduction paths can lead to Fano antiresonance manifesting as a dip in the linear conductance [23–24,27–28]. There are also reports on T-shaped carbon nanotube structures [29–30] and similar carbon devices engineered by attaching C_60_ buckyballs onto the sidewall of a single-walled carbon nanotube (carbon nanobud [31]). Many experiments showed that Kondo and Fano resonances can occur simultaneously [32–33].

**Figure 1:**
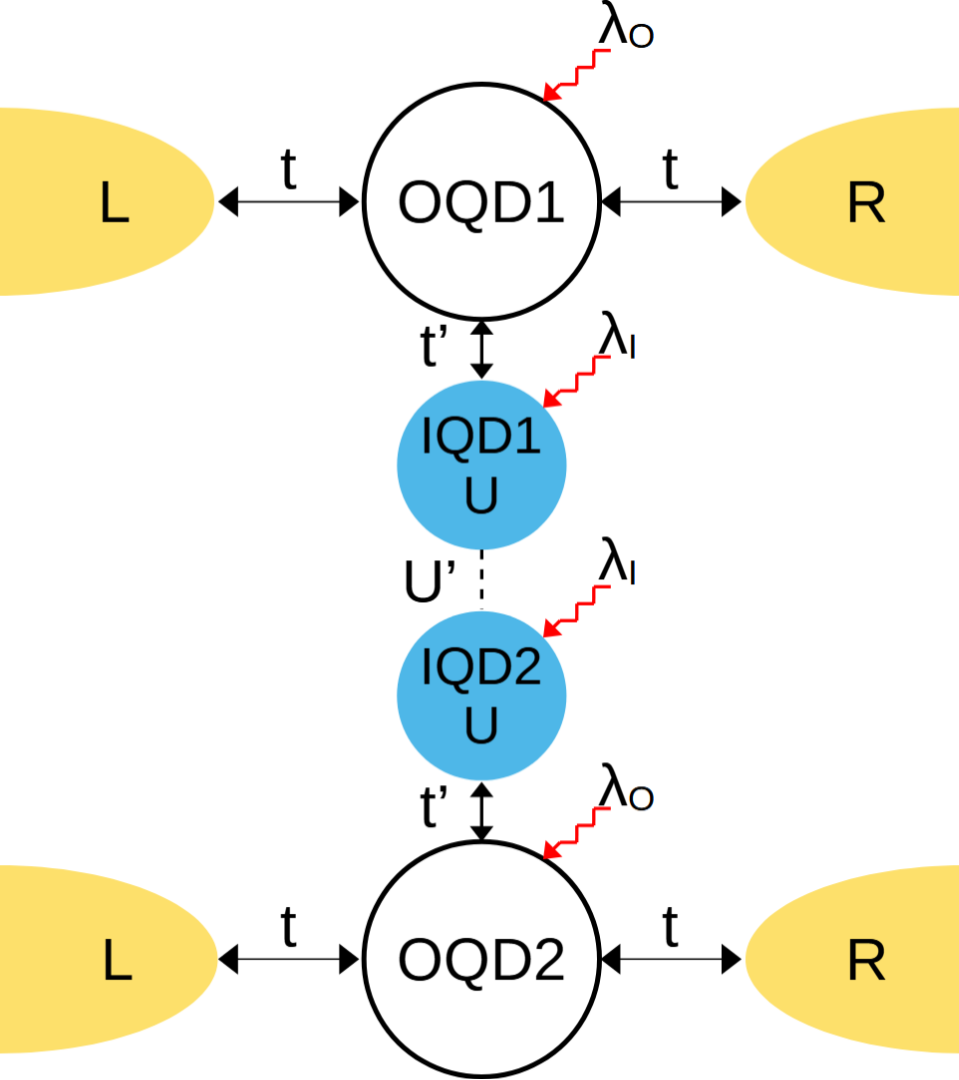
Schematic of capacitively coupled side attached quantum dots IQDs (DTQD) with electron-phonon coupling. The electrodes (L, R) are directly attached to the open quantum dots (vanishing interactions) OQD1(2). Phonons are coupled either to open (λ_O_ ≠ 0) or to interacting dots (λ_I_ ≠ 0).

Recently, there is also an increasing interest in nanoelectromechanical systems (NEMS) integrating electrical and mechanical functionalities [34–38]. Nanoelectromechanical systems utilizing localized mechanical vibrations have found applications in ultrafast sensors, actuators, and signal processing components. Of special interest are molecular systems because molecules due to their softness easily deform during tunneling processes, giving rise to excitation of local phonon modes. The polaronic transport through molecular systems has been recently studied in a number of papers [39–44]. Due to participation of localized phonons in single electron tunneling the phonon side bands appear in the spectral function of the dot. Interestingly, similar effects have been also observed in the rigid structures of semiconductor quantum dots embedded in a freestanding GaAs/AlGaAs membrane [44–48]. It has been shown that morphology manipulation of semiconductor QDs such as size, shape, strain distribution, or inhomogenities can influence the coupling strength of electron–phonon (e–ph) interactions [49]. The phononic effects appears not only in sequential tunneling, but also in the Kondo regime where vibrational sidebands have been also observed [45,50–54]. The interplay of electron–phonon coupling and Kondo effect has been also studied theoretically [55–60]. In the present paper we analyze the impact of electron–phonon coupling on both strong correlations and interference. We perform a discussion for quantum dots arranged in single (TQD) or double (DTQD) T-shaped geometries (see below Figure 1). Due to the quantum confinement, there may be also a confined phonon located in a single QD or molecule. Such a phonon interacts only with the electrons in the same QD. In the following considerations it is assumed that local phonons couple either to the open dots (OQDs) directly connected to the leads or to the dots attached to the interacting side (IQDs). The former type of coupling mainly influences interference conditions and also affects the correlations. In the latter type of coupling, only the correlations are modified. For phonons coupled to OQDs, a roughly exponential suppression of transmission and the occurrence of satellite Fano–Kondo dips is observed. These effects manifest only very weakly in the transmission through the open dot in the case when phonons couple to IQDs. However, they are reflected clearly in the density of states (DOS) of IQDs, but this is difficult to detect in transport experiments. The single T-shaped device decoupled from phonons is characterized by SU(2) symmetry, and the electron occupation ranges from zero to two. The double T-shaped device unperturbed by phonons has SU(4) symmetry with possible electron occupations from zero to four. Electron–phonon Holstein coupling, which we discuss, lowers the energy of doubly occupied orbitals relative to empty or single occupied orbitals. In consequence, the regions of occurrence of an even number of electrons in the system narrow down or completely disappear with the increase of the strength of e–ph interaction. Fluctuating spin doublets interfering with the wave propagating through a direct path give rise, through cotunneling processes, to the spin Fano–Kondo effect. For strong e–ph coupling, the charge ordered ground state of the system appears, and for special ranges of gate voltages and the corresponding values of e–ph coupling the charge Fano–Kondo effect occurs.

## Model and Formalism

We consider two types of T-shaped structures. The first is a single T-shaped system, in which one of the dots (noninteracting dot – OQD) is coupled directly to the leads and the second dot (interacting – IQD) is coupled indirectly through the open dot. The scheme presented in Figure 1 shows two capacitively coupled TQD systems (DTQD).

It is worth noting that an orbitally degenerate TQD system with equal intra- and interorbital interactions is equivalent to the DTQD we consider. This interpretation of DTQD systems is more adequate for molecular systems. Both side coupled dot systems, TQD and DTQD with Kondo resonances of interacting dots, have already been analyzed [22,24,61–64], but here we generalize these considerations focusing on the role of phonons in electron transport through these structures. Discussing the e–ph coupling in the introduced systems we consider three special cases: 1) local phonon modes are coupled solely to the open dots (*l* = 1), 2) local phonon modes are coupled only to the interacting dots (*l* = 2), and 3) single local phonon mode is equally coupled to both interacting dots (*l* = 3). The corresponding DTQD Hamiltonians representing the three mentioned e–ph types of coupling are written below:


[1]

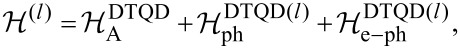



where 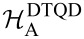 is the double dot or double orbital Anderson Hamiltonian for T-shaped geometry, which is written as:


[2]

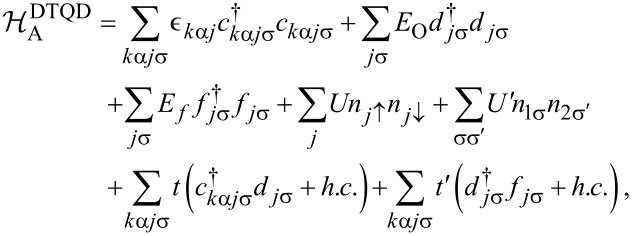



where the first term describes electrons in the electrodes and the next two terms represent electrons residing on the open (*d_j_*_σ_) and the interacting (*f_j_*_σ_) dots, respectively. *j* enumerates the upper (*j* = 1) and the lower (*j* = 2) TQD subsystems visualized on Figure 1 and α numbers left or right electrode. The terms parametrized by *U* and *U*’ describe intra- and interdot Coulomb interactions, respectively, with 
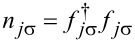
 denoting the occupation operators of IQDs. The last two terms stand for tunneling between the electrodes and open dots and between the dots. In case of no e–ph coupling intra- and interdot interactions are assumed to be equal (*U* = *U*’). 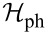 is the phonon Hamiltonian and 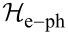 is the Holstein electron–phonon coupling term [65].


[3]

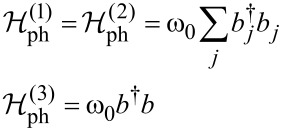



where ω_0_ is the frequency and *b_j_* is the annihilation operator of the localized phonon mode (we set ℏ = *k*_B_ = |e| = 1).


[4]

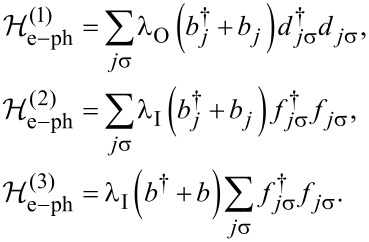



The dot Hamiltonian for the TQD system is


[5]

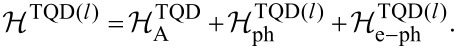



We do not write explicit forms of 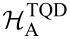, 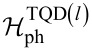, and 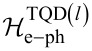 (*l* = 1, 2) since they only differ from Equations 2–4 by a lack of summation over the phonon modes and dots. In this case only a single phonon couples to electrons. The term describing the interaction between the dots parameterized by *U*’ does not appear either.

Following Lang and Firsov [66–67] the electron–phonon couplings in a DTQD can be eliminated by canonical transformations:


[6]

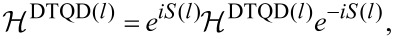



with


[7]

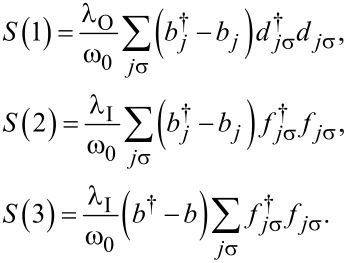



The new fermion (polaron) operators are 
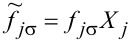
 and 
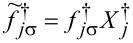
 with 
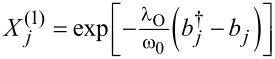
 (*l* = 1) and, similarly, for the coupling with interacting dots 
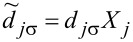
 and 
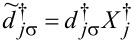
 with 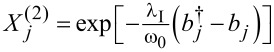 for *l* = 2 and 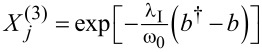 for *l* = 3.

The diagonalization [6] is exact if *t* = *t*’ = 0 or λ_O_ = λ_I_ = ∞. This transformation shifts the dots to the new equilibrium positions and in general changes the phonon vacuum. In the following we restrict in the expansion of 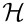 up to the terms λ^2^. In this sense, the results for stronger coupling should be viewed with caution, treating them only as a quantitative, preliminary insight into the problem. Higher commutator approximations improve accuracy of canonical transformation, but introduce numerical difficulties [68]. A very strong coupling regime is usually described starting from the infinite coupling solution and then performing perturbation expansion in terms of 1/λ [69]. Analogous unitary transformations decoupling the entanglement of electrons and phonons in TQD systems have the same form, but again without summing over index *j*. The DTQD Hamiltonians are transformed into 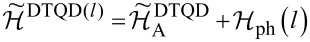. 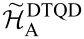 has the same form as 
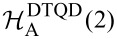
 (Equation 2), but with the old fermion operators replaced by new operators 
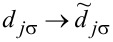
 for *l* = 1 or 
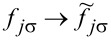
 for *l* = 2, 3 and the use of renormalized parameters. For *l* = 2, 3 the parameters *E*_f_ and *U* are shifted due to e–ph interactions by a renormalization constant 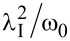, 
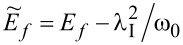
, 
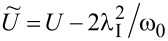
 (*l* = 2) and 
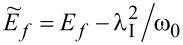
, 
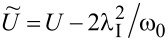
, 
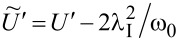
 (*l* = 3). As it is seen Holstein coupling lowers the energy of doubly occupied orbitals relative to single occupied or empty orbitals. For *l* = 1 e–ph interaction effectively shifts *E*_O_, 
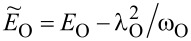
 and attractive phonon-induced interaction 
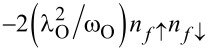
 appears. For *l* = 2, 3 hopping term between the open and interacting dot is also renormalized by a factor *X*, which describes the effect of the phonon cloud accompanying the hopping process 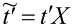. For *l* = 1 both hopping integrals are renormalized 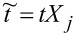 and 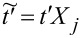. Assuming the relaxation time of phonons to be much shorter than the time of electron transport through the dot (antiadiabatic limit) allows one to consider the phonon subsystem as being approximately in thermal equilibrium. The phonon operator *X* can be then replaced with its expectation value [67] ⟨*X*⟩ = exp[−(λ^2^/ω_0_)(*N*_ph_ + 1/2)] with λ = {λ_I_,λ_O_}, depending on the analyzed case and *N*_ph_ is given by the Bose–Einstein distribution. In this approach the electron and phonon dynamics become decoupled. This approximation, which is widely used in literature [49,58–59,70–72], predicts the exponential suppression of the tunneling amplitudes (Franck–Condon (F–C)-type suppression). Using the form of new fermion operators obtained in Lang–Firsov transformation it is easy to show that the electron Green's function of the dot can be decoupled as [73–74]:


[8]

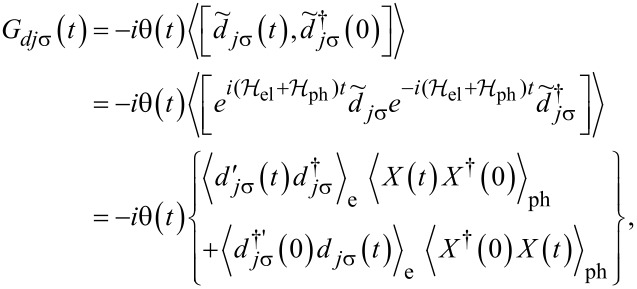



where 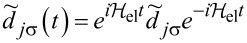, 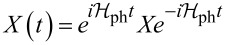, and 
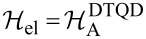
 or 
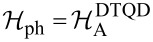
. The renormalization factor due to e–ph interaction is evaluated as [67]:




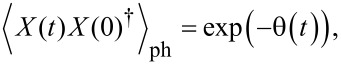




where


[9]

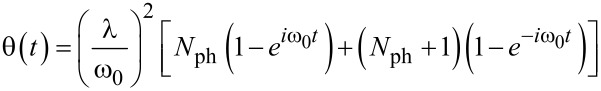



For the zero-temperature case we are discussing a situation where ⟨*X*(*t*)*X*^†^(0)⟩ is reduced to


[10]

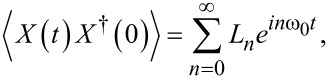



where


[11] $$L_{n}=\frac{1}{n!}{\left(\frac{\lambda }{\omega_{0}}\right)}^{2n}e^{-{\left(\frac{\lambda }{\omega_{0}}\right)}^{2}}$$

This approximate formula will be used by us in the following, because we are interested only in the low-temperature transport. The Fourier transforms of the retarded Green’s functions of the dots to which the phonons are attached is then given by


[12]

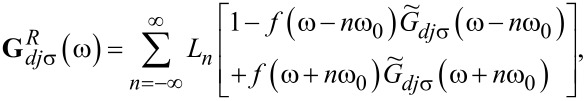



where *f*(ω) is a Fermi distribution function and the retarded dressed Green’s functions 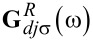 are the functions corresponding to the Hamiltonian in Equation 6 for *l* = 2, 3. An analogous expression to Equation 11 holds for 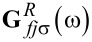 (the Hamiltonian in Equation 6 with *l* = 2, 3). To find the dressed Green’s functions and consequently discuss correlation effects we use the finite-U slave boson mean field approximation (SBMFA) of Kotliar and Ruckenstein [75–76], which we apply to the effective polaron Hamiltonian of DTQD (Equation 6) or the analogous Hamiltonian of a TQD. For the latter case a set of auxiliary bosons *e*, *p*_σ_ and *d* projecting onto empty, single occupied and doubly occupied states of the interacting QD are introduced. In the DTQD system, in addition to the operators *e*, *p*, and *d*, we also introduce *t* and *f* SB operators representing triple and quadruple dot fillings, respectively. The single occupation projectors *p_j_*_σ_ in this case are additionally labeled by the orbital or interacting dot index *j*. A similar notation applies for the triple occupancy boson *f_j_*_σ_, but this time the index *j* indicates an interacting dot or orbital, which is not fully occupied (occupation of a hole). Six {*d_i_*,*d*_σσ′_} operators project onto (↑↓,0) and (0,↑↓) for (*d_j=_*_1_*_,_*_2_) and (↑,↑),(↓,↓),(↑,↓),(↓,↑) for (*d*_σσ′_) [77]. To eliminate unphysical states we introduce the following constraints: the completeness relation for the slave boson operators and the condition for the correspondence between fermions and bosons. These restrictions can be enforced by introducing Lagrange multipliers (Δ’,Δ*_j_*_σ_) and supplementing the effective slave boson Hamiltonian by the corresponding terms. For brevity we write only the SBMFA Hamiltonian of a DTQD for *l* = 2.


[13]

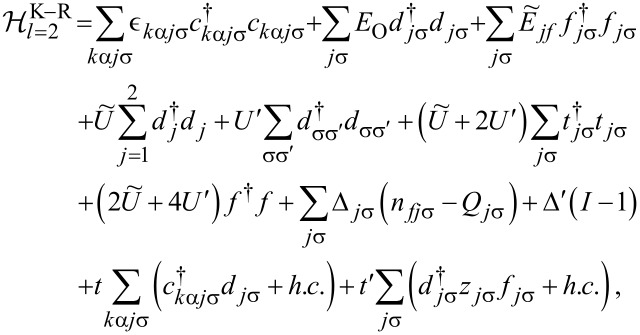



where the pseudofermion operators *f_j_*_σ_ are defined by




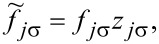




and




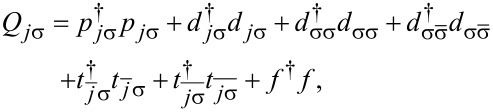







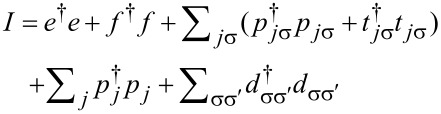




and




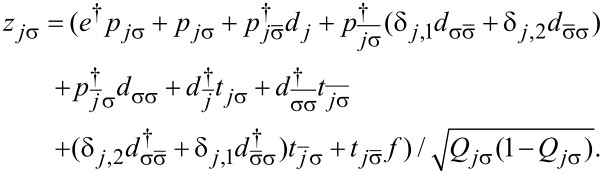




*z_j_*_σ_ renormalizes interdot hoppings and dot-lead hybridization. The stable mean field solutions are found from the minimum of the free energy with respect to the mean values of boson operators and Lagrange multipliers. For the high-symmetry coupling cases discussed, where two Kondo dots play identical roles, the number of independent bosons is reduced to six (*e*, *p*, *d*, *d*’, *t*, and *f*) and two Lagrange coefficients are sufficient (Δ and Δ’). In the SBMFA procedure the problem of strong interactions is formally reduced to the effective free electron model with renormalized hopping integrals and dot energies. SBMFA best describes systems close to the unitary Kondo limit. However, due to its simplicity, this approach is also often used in analysis of the linear conductance of systems with weakly broken symmetry giving results in a reasonable agreement with experiment and with numerical renormalization group calculations [67]. Mean field approximation best works at low temperatures, where it is justified to neglect fluctuations of the boson fields. We restrict our analysis to equilibrium, so inelastic transport produced by e–ph interaction can be neglected. We consider conductances through the upper TQD subsystem (*j* = 1) and through the lower subsystem (*j* = 2). They are separately experimentally accessible. According to the derivation based on the nonequilibrium Green function formalism [78] linear conductances of the wires with embedded open dots are given by a Landauer-type formula:


[14]

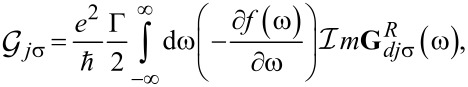



where Γ is the coupling strength to the electrodes (for the rectangular density of states of electrodes 1/2*D* for |*E*| *< D*, Γ = π*t*^2^/*D*). For the case when phonons are coupled to the open dot Γ should be replaced by 
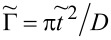
, 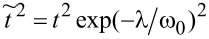. **G***_djσ_* denotes the Green’s function of OQDj, which according to Equation 12 can be approximately expressed as




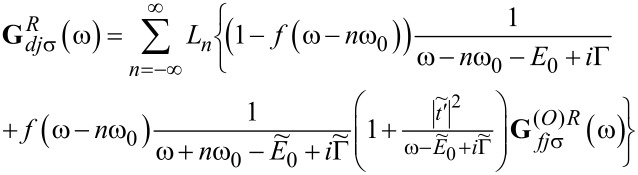




with




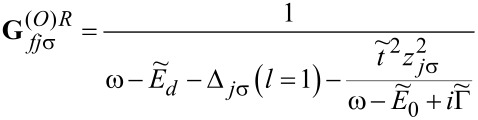




for *l* = 1 and




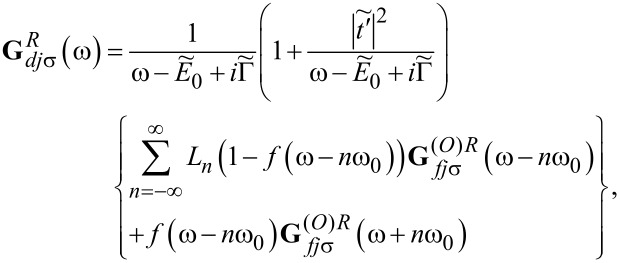




for *l* = 2, 3 with


[15]

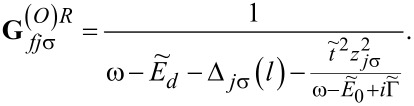



## Results and Discussion

Before we present numerical results, let us look at the energy scales we are going to discuss. Typical values of the bandwidths of the metallic organic wires are several hundreds of millielectronvolts [79–80]. The charging energy parametrized by *U* can be inferred from the size of the Coulomb diamonds. It increases with the decrease of the size of the QDs. Typically, for molecular and semiconducting QDs, it ranges from several to tens of millielectronvolts [81–84]. Coupling between the QD and reservoirs Γ can be estimated from the width of Coulomb peaks and ranges from hundreds of microelectronvolts up to several millielectronvolts in the weak-coupling regime considered by us [14,82]. Phonon energies of molecular systems range from microelectronvolts up to millielectronvolts [85–86]. The experimental values for the electron–phonon coupling strength depend on the specific setup. All ranges, that is, weak, intermediate, and strong coupling, are accessible. As an example, data for carbon systems are given: The experimental results for suspended carbon nanotube QDs showed an average value of strong coupling λ ≈ 1.7 [48]. For fullerene C_60_ intermediate coupling is observed λ ≈ 0.5 [45], and for different C_140_ samples λ ranges between 0.1 and 4 [87–88]. In this section we present numerical calculations illustrating the effect of phonons on interference and electron correlations and we show how this effect is reflected in the conductance values. Throughout this paper we use relative energy units choosing *D*/50 as the unit, where *D* is the electron bandwidth of the leads. We assume the following values of parameters: the bare Coulomb integrals in the absence of phonons *U* = *U*’ = 3, coupling strength to the electrodes in the range Γ ∈ ⟨0.05,0.1⟩, and phonon energy ω_0_ = 0.5. The Fermi energy of the leads is taken as *E*_F_ = 0. All results have been calculated for the strong e–ph coupling limit (λ_O_, λ_I_ *>* Γ) and are compared with the cases without phonons (λ_O_ = 0, λ_I_ = 0). The shape of the bare transmission lines of the considered T-shaped systems is determined by the Fano parameter *q* = *E*_O_/Γ, which can be tuned by gate voltage. For *q* = 0 interference between the ballistic channel through the open dot and the Kondo resonant channel leads to the symmetric dip structure with vanishing transmission for SU(2) symmetry (destructive interference, Fano–Kondo antiresonance) and to half-reflection for SU(4). This is a consequence of π/2 or π/4 phase shifts for SU(2) or SU(4) symmetries, respectively. A complete reflection for SU(4) symmetry occurs for *q* = −1 (destructive interference) and full transmission for *q* = 1 (constructive interference). The SU(2)-symmetric T-shaped system exhibits unitary transmission for *q* = |∞| and in this limit it is roughly equivalent to the embedded QD [62].

### Single T-shaped quantum dot structure

**Figure 2:**
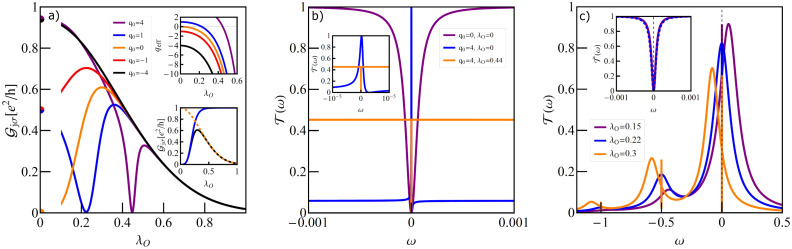
(a) Partial conductance of a single T-shaped system TQD with a phonon mode coupled to a OQD plotted for different values of Fano factor *q*_0_ (*E*_f_ = −1.5). The inset at the top of panel (a) shows the effective Fano factor as a function of λ_O_. The bottom inset illustrates the impact of Franck–Condon-type suppression. The orange dashed line presents the F–C factor, the black solid line shows the dependence of conductance on λ_O_ for *q*_0_ = 0, resulting from both renormalization of *q* and F–C suppression, and the blue solid line shows the effect on conductance of only phonon-induced renormalization of *q*. (b) Selected transmissions of TQD for λ_O_ = 0, *q*_0_ = 0, *q*_0_ = 4 and for λ_O_ = 0.44, *q*_0_ = 4. (c) Transmission of the TQD in a wide range of energy ω showing the traces of phonon modes around *n*ω_0_. In the inset the normalized transmission for λ_O_ = 0.22 around ω = ω_0_ is imposed on the normalized transmission around ω = 0.

Figure 2a illustrates the dependence of the conductance on the e–ph coupling strength of the TQD for the case when a local phonon is coupled to the open dot. The curves are plotted for different unperturbed Fano parameters. For vanishing coupling (λ_O_ = 0) interplay of Kondo correlations and interference results in a Fano–Kondo antiresonance for *q* = 0 (Figure 2b) and, consequently, zero conductance is observed. For *q* ≠ 0 Fano–Kondo resonances are asymmetric and the dip does not enter the Fermi level and the corresponding conductances are finite. For *q*→∞ the resonance evolves into a Lorentzian peak at *E*_F_ [89].

**Figure 3:**
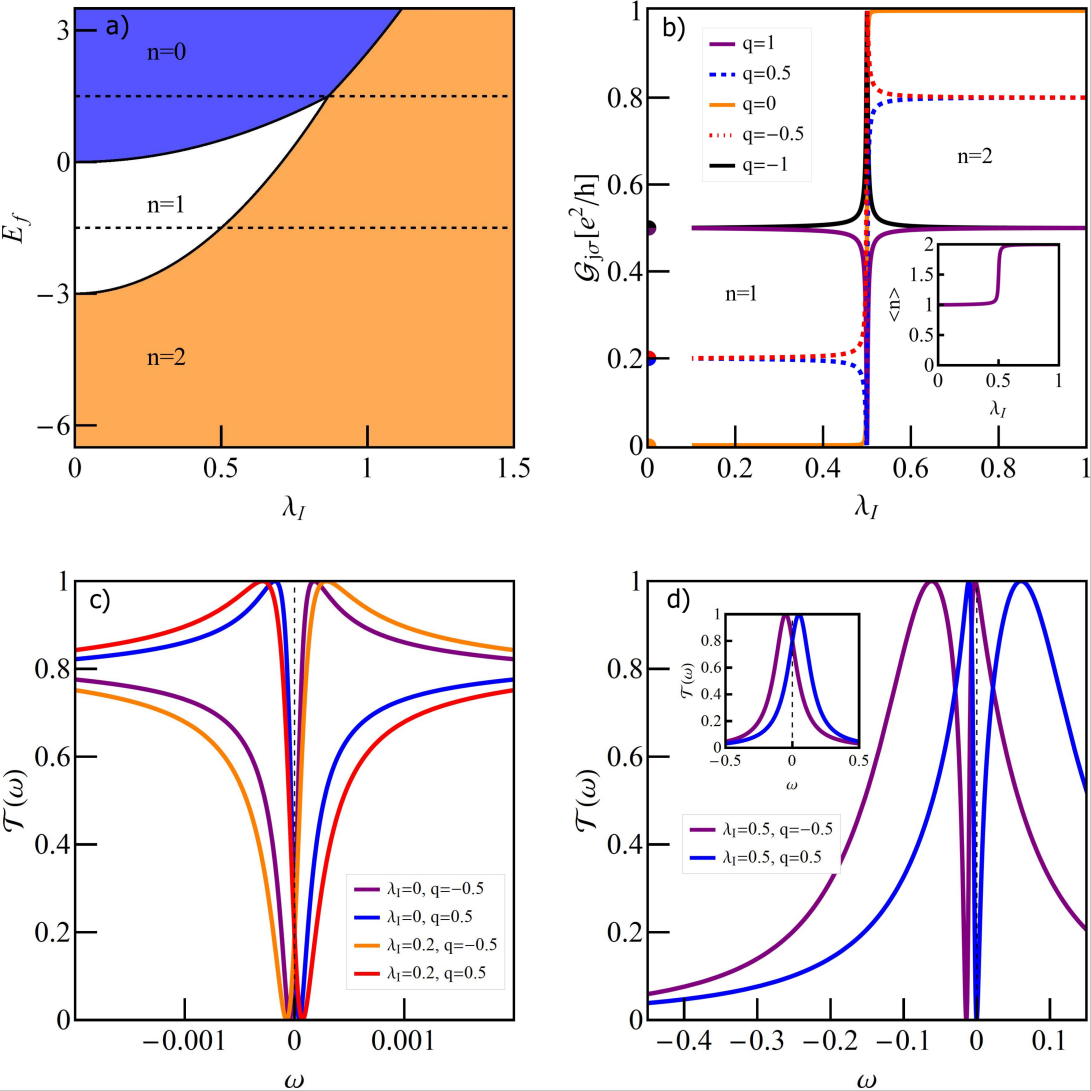
(a) Charge stability diagram of a TQD with a phonon coupled to the IQD as a function of gate voltage and e–ph coupling λ_I_. The dashed horizontal lines show the cross sections for which we present conductance curves (Figures 2–4). (b) Partial conductance of the TQD as a function of λ_I_ for several values of the Fano factor (*E*_f_ = −1.5), the inset shows the occupation of the IQD. (c, d) Transmissions for *q* = ±0.5 and (c) λ_I_ = 0, 0.2 or (d) λ_I_ = 0.5, and λ_I_ = 0.8 (inset of panel (d)).

**Figure 4:**
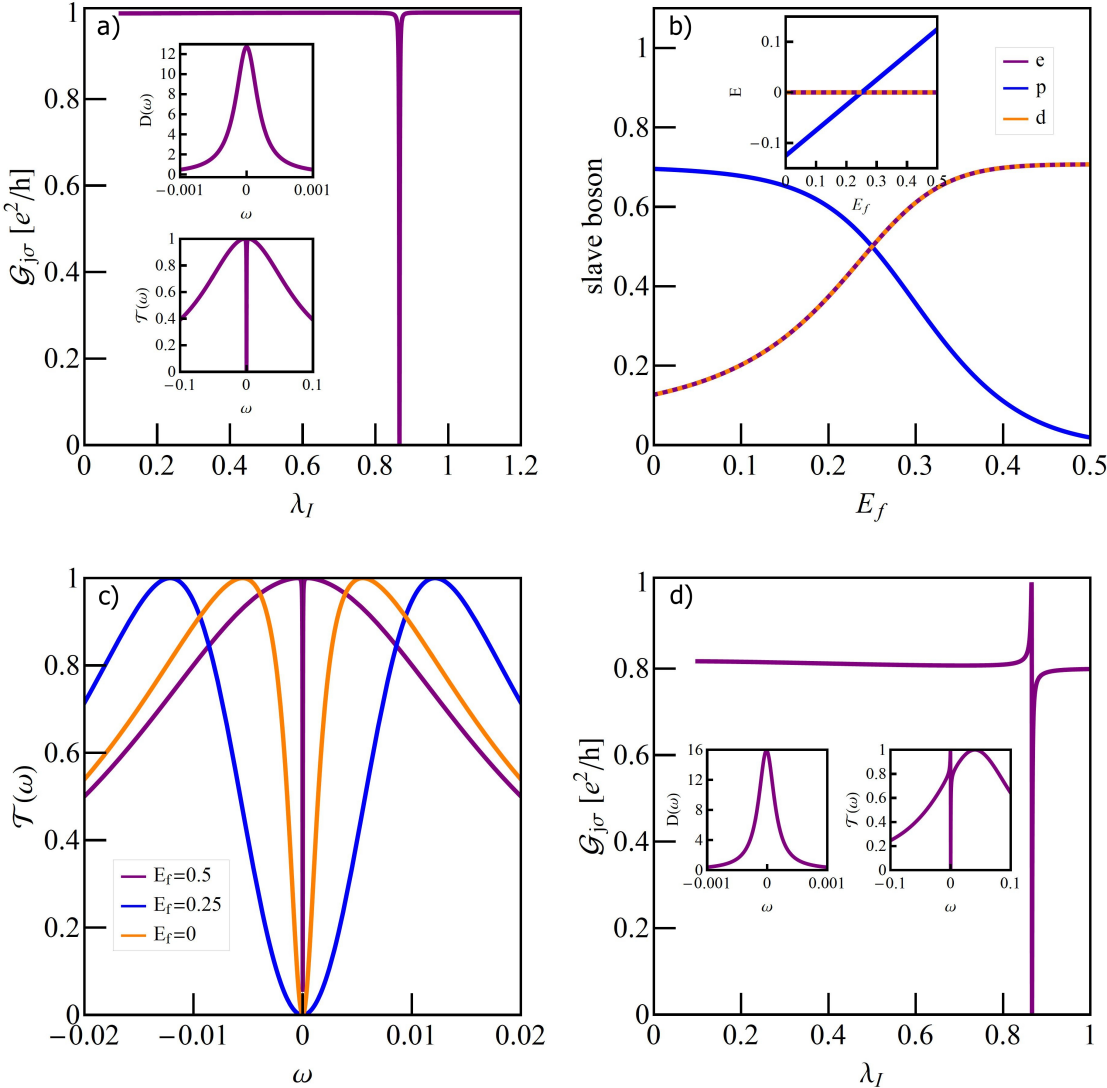
(a) Partial conductance of TQD for *E*_f_ = 0.5 with visible effect of charge Kondo resonance at λ_I_ = 0.43. Top inset shows density of states of IQD and bottom one transmission in charge Kondo state. (b) Gate dependence of slave bosons with λ_I_ on the line of e-d degeneracy. (c) Several representative transmissions for different gate voltages. (d) Partial conductance for *E*_f_ = 0.5 and *q* = 0.5 together with DOS of IQD and transmission at the Kondo point.

**Figure 5:**
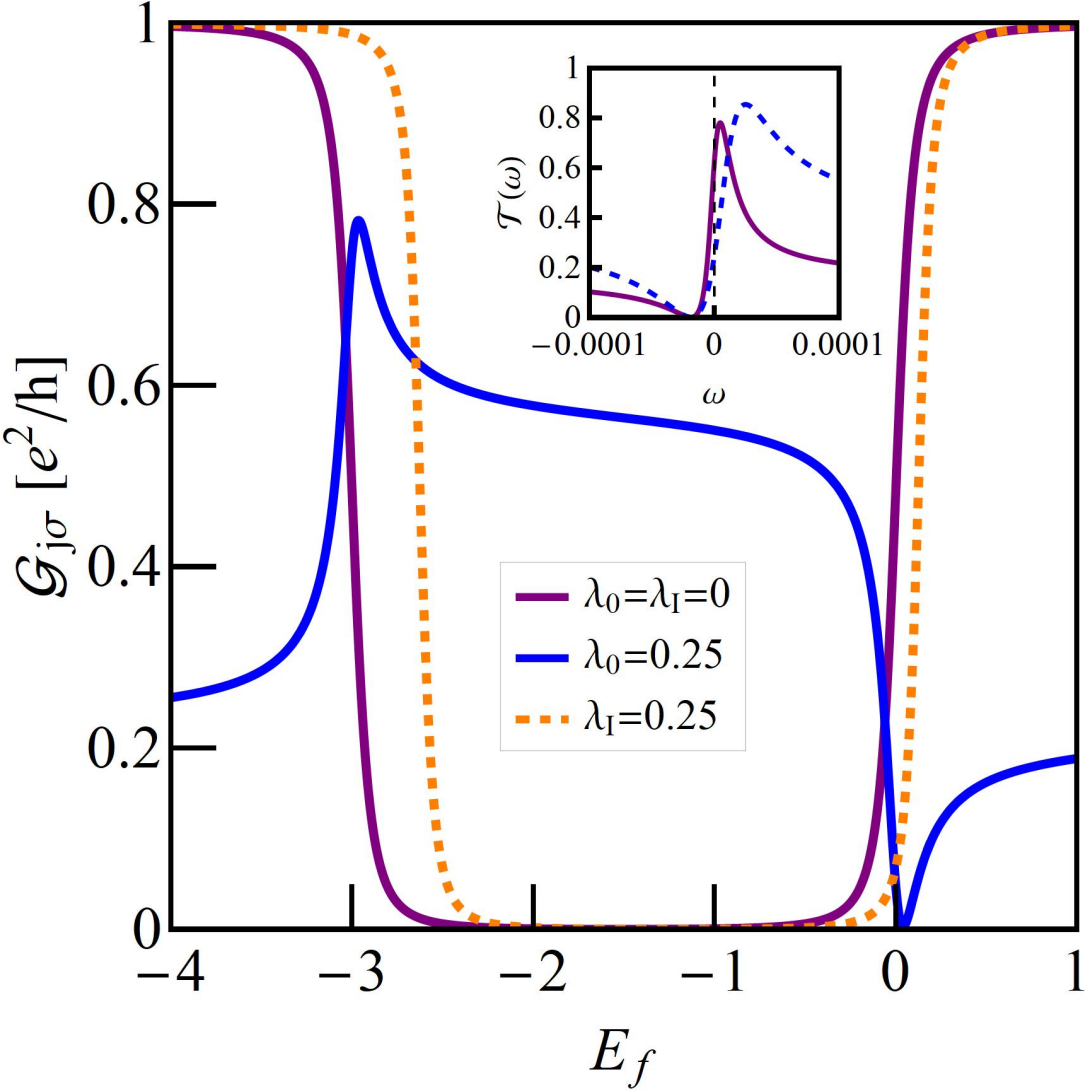
(a) Comparison of gate voltage dependencies of the TQD conductance with a phonon coupled to the IQD (symmetric orange broken line) or OQD (asymmetric blue line) with TQD conductance decoupled from the phonons. The inset presents the transmission for λ_O_ = 0.25 for *E*_f_ = −0.2 (dashed blue line) and *E*_f_ = −2.8 (solid purple line).

It is already partially visible in the transmission for *q* = 4 that λ_O_ = 0 (Figure 2b) and the conductance reaches nearly unitary limit. Looking at the transmission for *q* = 4 and λ_O_ = 0.44, where the effective Fano parameter vanishes, it is seen that the symmetric antiresonance is rebuilt, but transmission is suppressed due to e–ph coupling. It can be seen that the coupling with phonons modifies the interference conditions. The effective Fano parameters *q*_eff_, presented in the upper inset of Figure 2a decrease with increasing coupling. *q*_eff_ are determined by the coupling-dependent effective site energy of the open dot 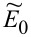 through polaron shift and by the phonon-dependent hybridization strength 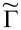, which changes according to the Franck–Condon factor, exp[−(λ_O_/ω_0_)^2^]. The lower inset Figure 2a shows the examples of conductance dependence on λ_O_ with the inclusion or neglect of the F–C factor. The exponentially decreasing line illustrates the pure Franck–Condon suppression (conductance for *q* = ∞). It is seen that for small values of *q* this suppression starts to play the decisive role for large values of coupling. For small values of e–ph coupling linear conductance increases for *q* ≤ 0, with the increase of λ_O_ and decreases for *q >* 0. The observed decrease of conductance for strong e–ph coupling is dictated by F–C suppression. The zeros of conductance correspond to *q*_eff_ = 0. Fano–Kondo resonance is also influenced by phonons through the change of Kondo correlations resulting mainly from the renormalization of interdot hopping *t*’ and transmission variations on the open dot induced by the changes of electron–phonon coupling. Figure 2c shows examples of the transmission for *q*_0_ = 1 for the selected values of λ_O_. The main peak and the satellites move towards lower energies with the increase of e–ph coupling, which corresponds to the phonon-induced shift of *E*_O_. The height of the main peak decreases according to the F–C factor. The dips observed for ω = 0 for the main peaks (see inset) and for ω = *n*ω_0_ for the satellites, exhibit Fano shape corresponding to a given *q*_eff_. For λ_O_ = 0.22, where *q*_eff_ = 0, the line is symmetric. It is worth to stress that the widths of the dips in the main peak are the same as in the satellites, which proves that both reflect the same phenomenon, that is, Kondo resonance on the interacting dot. We have also checked that the satellite peaks follow the same temperature dependence as the main Kondo peak. So far we have presented the conductance for occupation *n* = 1 only. The following figures (Figures 3–5) refer to a TQD system with phonons attached to an interacting dot.

The charge stability diagram of a TQD as a function of IQD energy *E*_f_ and e–ph coupling constant λ_I_ is presented on Figure 3a. It is seen that transfers between regions of different occupations are possible either by changing the gate voltage (change of the dot single particle energy *E*_f_) or by modification of electron–phonon coupling. Polaron-induced suppression of charging energies shifts the Coulomb blockade boundaries and narrows Coulomb valleys. Possible transitions are *n* = 1→*n* = 2, *n* = 0→*n* = 1→*n* = 2 and *n* = 0→*n* = 2. Let us first concentrate on the phonon-induced 1→2 transition (Figure 3a). For *n* = 1 SU(2) Kondo resonance forms on the IQD. Due to the interference with the wave propagating through the OQD Fano–Kondo resonance appears determined by the value of *q*, which in this case does not change with the strength of the e–ph coupling λ_I_. In consequence, Fano–Kondo conductances remain almost unchanged in the whole range of single occupation. For *q* = 0 antiresonance blocks the linear transport. The asymmetry of the Fano line does not change with λ_I_, but the width of the dip does due to phonon-induced renormalization of interdot hopping *t*’ and polaron shifts of site energy and Coulomb interaction of the IQD (compare transmissions for λ_I_ = 0 and λ_I_ = 0.2 (Figure 3c)). The total Fano–Kondo conductance takes the value of zero for *q* = 0 (antiresonance) and *e*^2^/*h* for *q* = ±1. For λ_I_ = 0.5 a transition 1→2 to the new charge state takes place. The shapes of the transmission lines are combined effect of charge transition and interference. Obviously the widths of the dips appearing here are wider than for Fano–Kondo resonances. *T*(*E*_F_) reaches one for *q <* 0 and it takes the value of zero for *q >* 0. This is illustrated in Figure 3d for the exemplary case of *q* = ±0.5, but these limits are valid for any value of *q*. This behavior is reflected in the conduction by its jump to the unitary limit or in its complete suppression in the transition point. For λ_I_ *>* 0.5 occupancy increases to *n* = 2 and the fully occupied IQD stops affecting transport. Transmission in this range does not result from interference and the observed dependence on *q* only reflects the dependence on the site energy of the open dot *E*_O_. The widths of the lines are now determined by unperturbed dot–lead coupling (inset of Figure 3d). The next picture (Figure 4) presents direct transition in a TQD from the empty into the double occupied state 0→2 for *q* = 0 with a phonon coupled to interacting dot.

An empty (λ_I_ *<* 0.43) or fully occupied IQD (λ_I_ *>* 0.43) does not affect transport. The electron is transported through the OQD in these ranges with the probability amplitude *T*(*E*_f_) = 1. At λ_I_ = 0.43 the effective 0→2 charge fluctuations (pseudospin fluctuations) lead to the formation of charge Kondo resonance on the interacting dot, the interference of which with the wave propagating through an open dot causes the charge Fano–Kondo resonance to occur. This is reflected by the occurrence of the conductance dip. The resonance peak at the interacting dot and the corresponding symmetric dip of transmission are shown in the insets. They are considerably narrower than spin Fano–Kondo dips from Figure 3c. Figure 4b shows boson amplitudes for different dot energies *E*_f_ and e–ph coupling λ_I_ chosen such that empty and double occupied states degenerate. In the charge Kondo state, the low-energy excitations are charge fluctuations and there is a large gap for spin fluctuations. In SB language it means that equal *e* and *d* amplitudes are close to the value 
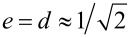
 corresponding to the transport unitary limit and *p*_σ_ amplitudes are small. As it is seen this condition is fulfilled for *E*_f_ = 0.5 for which dependencies from Figure 4a are drawn. For lower values of *E*_f_ the role of spin fluctuations increases as can be seen from the increase in *p*_σ_ at the expense of *d* and *e* amplitudes. This fact is also demonstrated by the energy dependency of the states corresponding to *n* = 0, 1, 2 plotted as function of *E*_f_ in the inset of Figure 4b. This is also reflected in the broadening of the resonance line on the open dot as shown in Figure 4c. The presented transmissions correspond to charge Fano–Kondo state, mixed valence, and spin Fano–Kondo state, respectively. For *E*_f_ = *U*/2 all four states degenerate and below this value spin fluctuations gradually take over the leading role. In the region of low values of *e* and *d* amplitudes the spin Kondo resonance will form. This time the amplitudes of *p*_σ_ operators take values close to 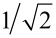. Figure 4d shows examples of perturbed charge Fano–Kondo effects for asymmetric cases *q* = 0.5. As expected resonance lines on the IQD are wider in this cases. Since for *n* = 0 and *n* = 2 the interacting dot is decoupled from the transport path, the presented conductance in these ranges is fully determined by the position of *E*_O_, that ism by the value of *q*. Figure 5 presents the gate dependence of conductance for the discussed cases. For *q* ≠ 0 interference introduces an asymmetry of conductance with respect to e–h symmetry point *E*_f_ = −*U*/2. Since phonon coupling with an OQD changes interference conditions, the symmetric conductance for *q*_0_ = 0 becomes asymmetric for λ_O_ ≠ 0. In contrast, coupling with an IQD (λ_I_ ≠ 0) maintains symmetry.

**Figure 6:**
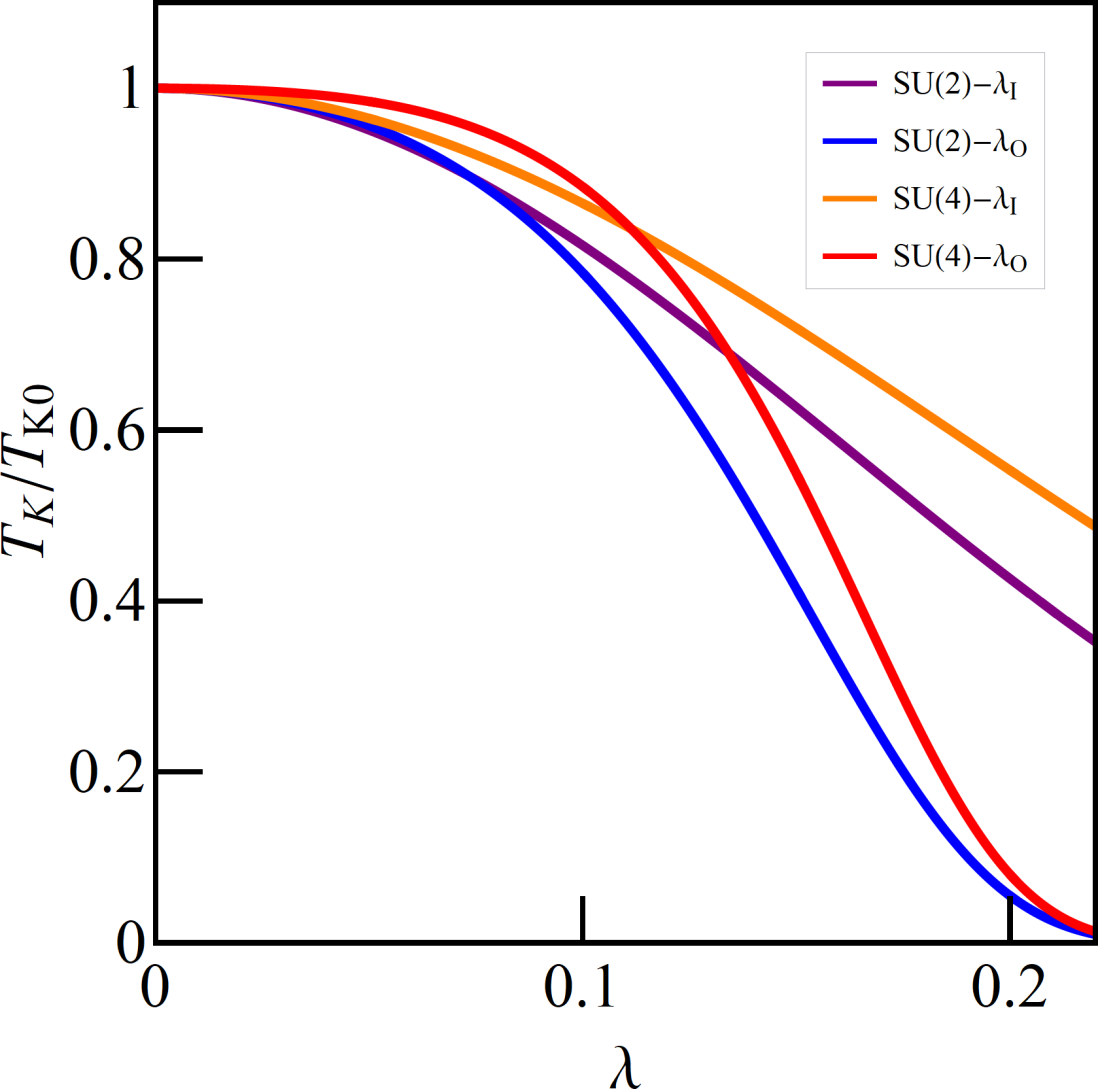
Relative Kondo temperatures vs e–ph coupling strength of TQD and DTQD coupled to the single phonon mode (*E*_f_ = −1.5).

Figure 6 compares the dependency of the Kondo temperature on the strength of e–ph coupling for the phonon mode coupled either to the open dot or to the interacting dot of the TQD structure (SU(2) symmetry) and of the DTQD structure with a single phonon mode (SU(4) symmetry). The latter problem is discussed in the next section. It is seen that, in both cases, the decrease of *T*_K_ with the increase of λ is faster for the case of a phonon attached to the open dot than for the case in which a phonon is connected to the interacting dot. This indicates that among the polaron effects, the exponential decay of the hopping integrals has a dominant impact on the lowering of the Kondo temperature. Before discussing the results for a DTQD let us first comment on the SU(4)-symmetric case to which we refer in the analysis below. As already mentioned in the introduction, many experimental facts [10,13–17] are attributed to the observation of this symmetry. Although the fully symmetric double dot system is only an idealization, some studies suggested [90], that emergent low-energy SU(4) symmetry can be restored also for slightly asymmetric systems by appropriately adjusting the gate voltages. More recent analysis based on NRG calculations [91] showed however, that for *U*’ *< U* the restoration of symmetry in the low-energy range might only happen if the interdot interaction is greater than the half bandwidth of the leads, which is experimentally unrealistic. In our considerations, the equality of all pure Coulomb interactions is not crucial, because by changing the electron–phonon coupling they differentiate or equalize anyway. We assume equal values of *U* and *U*’ only for clarity of presentation. Certainly the problem whether the emergent SU(4) state could be reached through e–ph coupling is interesting in itself, but this would require a more detailed analysis.

### Double T-shaped quantum dot structure

**Figure 7:**
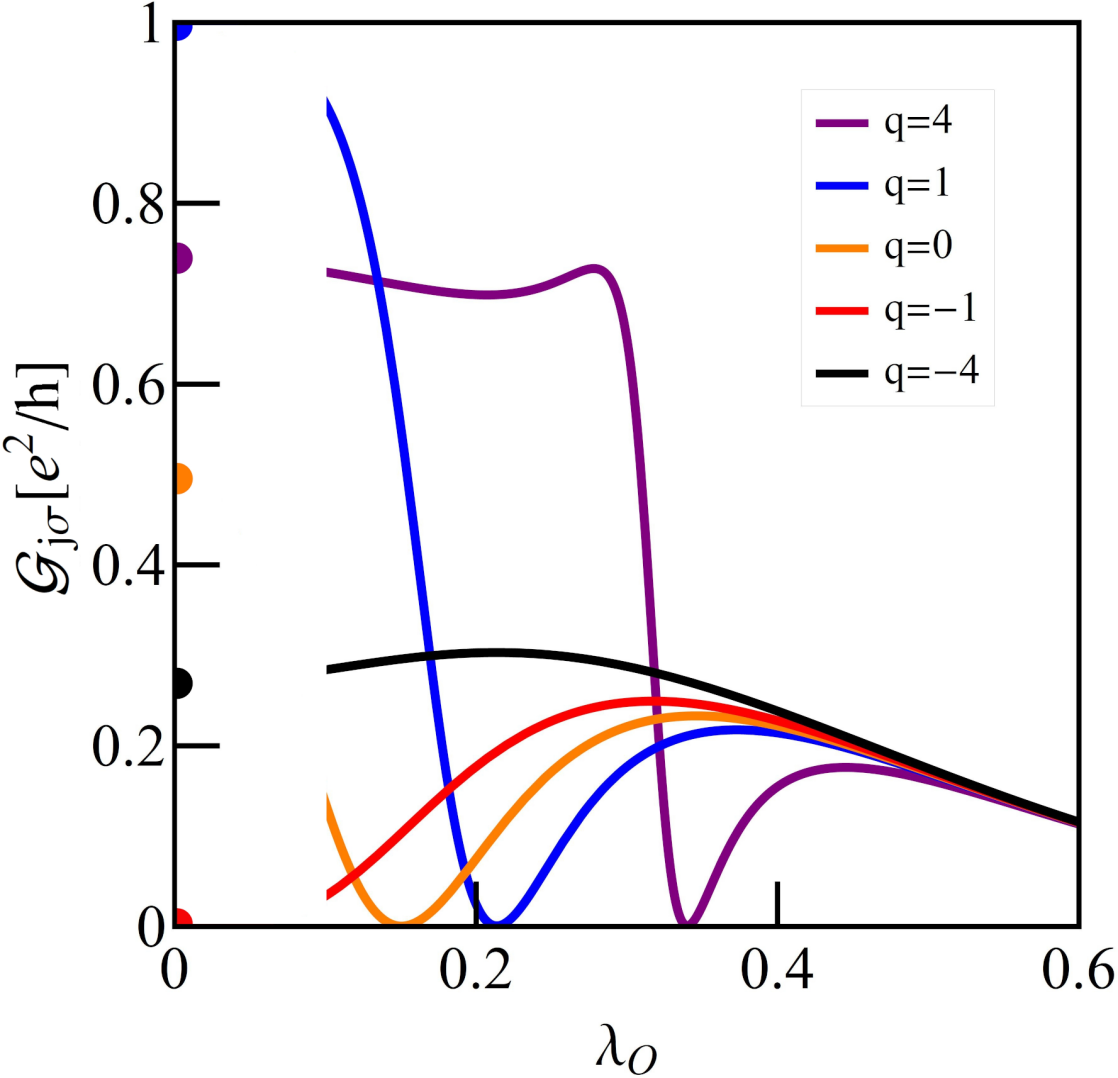
Partial conductance of a DTQD with phonons coupled to OQDs as a function of λ_O_ for different Fano factors for *E*_f_ = −1.5.

Figure 7 presents the impact of phonons coupled to the open dots on the conductance of a DTQD system. For λ_O_ = 0 Fano–Kondo resonance for *q* = 0 is revealed through half reflection, for *q* = −1 antiresonance is formed and the conductance drops to zero, and for *q* = −1 unitary conductance is observed. The observed dependencies are the combined effects of phonon-induced renormalization of Fano factors *q*_eff_ (change of the interference conditions) and Franck–Condon suppression. As mentioned earlier, the latter factor plays a crucial role for large values of e–ph coupling, where all the conductance curves for different *q*_0_ start to converge. The zeros of conductance are achieved when *q*_eff_ reaches −1 (destructive interference). For *q*_eff_ = 1 the phenomenon of constructive interference occurs (unitary transmission) and for *q*_eff_ = 0 half reflection is observed.

**Figure 8:**
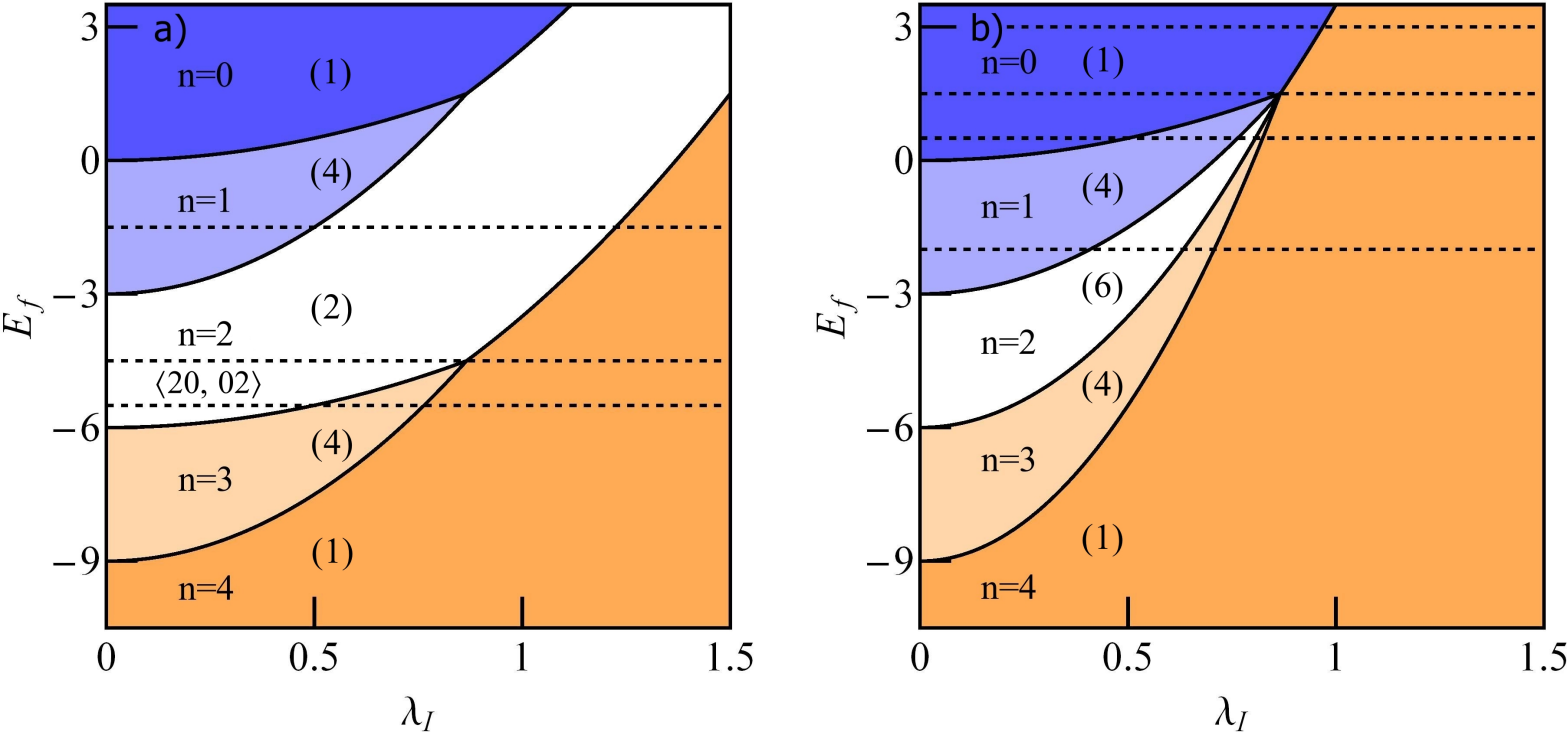
(a) Charge stability diagram of a DTQD with phonons coupled to the interacting dots as a function of gate voltage and e–ph coupling λ_I_. Apart from occupation numbers also the corresponding degeneracies of the ground states are given in the brackets. The dashed horizontal lines indicate the cross sections for which we present conductances below. (b) Charge stability diagram of a DTQD with single phonon coupled to both interacting dots.

Figure 8 presents charge stability maps of a DTQD when two phonons are attached to the interacting dots (Figure 8a) and when a single phonon is coupled to both IQDs (Figure 8b). The map is plotted versus the energy of the interacting dot *E*_f_ and the e–ph interaction constant λ_I_. Degenerations of the ground states are also marked on the map. Two triple-charge points, that is, points of coexistence of states characterized by three different charges, are visible on the map in Figure 8a (*n* = 0, 1, 2 and *n* = 2, 3, 4). In each of these points seven states are degenerate. Horizontal dashed lines indicate the cross sections for which the plots of conductances are presented below. An interesting feature of the map is the occurrence of the quadruple-charge point and the possibility of phonon-induced 0→4 charge transition. At the quadruple-charge point all sixteen double-dot states are degenerate.

**Figure 9:**
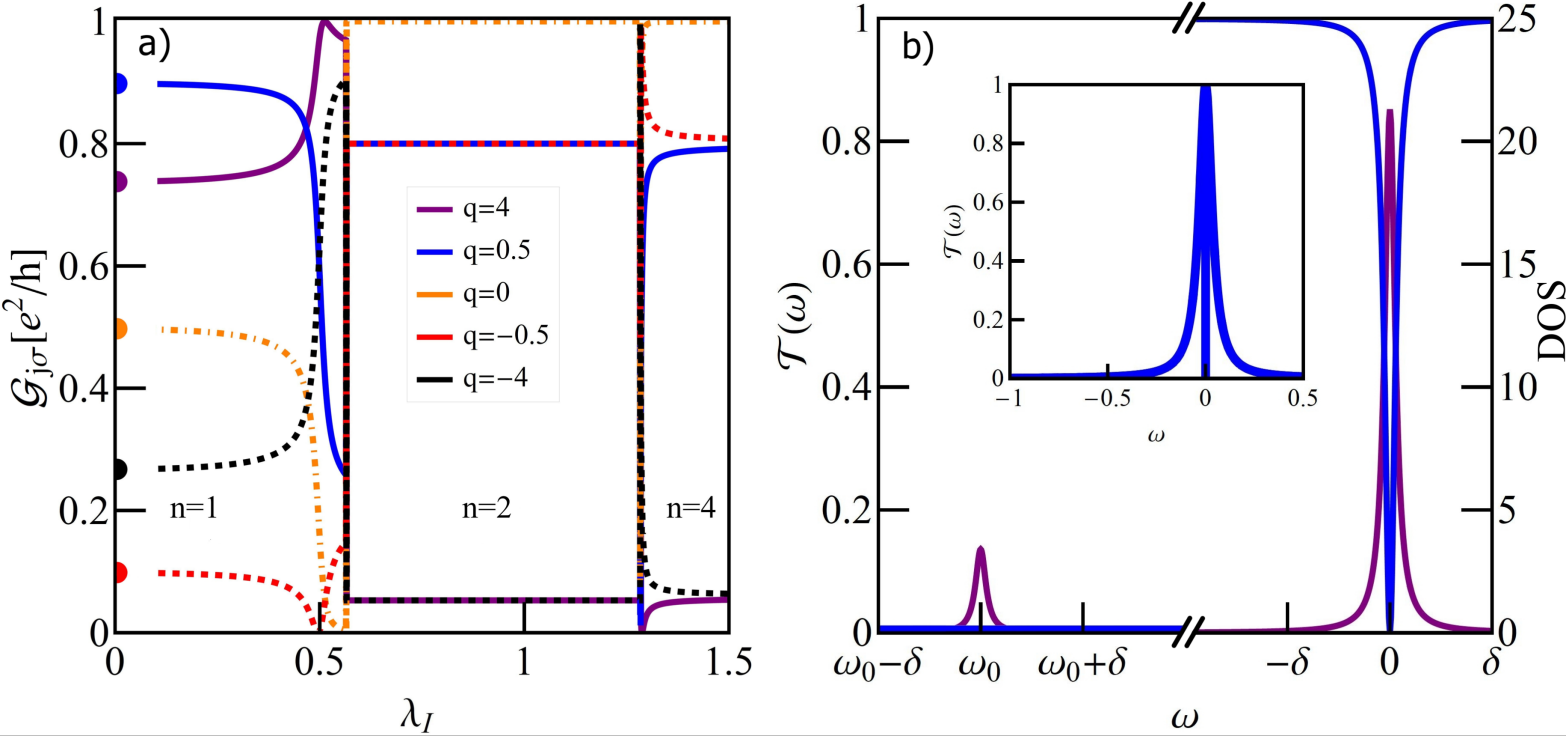
(a) Partial conductance of a DTQD with a pair of phonons as function of e–ph coupling λ_I_ for *E*_f_ = −1.5, for different Fano factors. (b) Density of states and transmission for the DTQD system with e–ph coupling λ_I_ = 0.2, *E*_f_ = −1.5, and δ = 0.001. Phonon modes are reflected in the DOS by the appearance of satellites, but no traces of them are visible in transmission. The full shape of transmission is shown in the inset.

Figure 9a shows the conductance vs λ_I_ for *E*_f_ = −1.5 (charge transition path *n* = 1→*n* = 2→*n* = 4). For λ_I_ = 0, the DTQD exhibits SU(4) symmetry and interference between the paths through the IQDs, where Kondo resonance is formed. Direct paths through the OQDS leads to the appearance of Fano–Kondo resonance where the conductance is determined by the Fano parameter *q*. For *q* = 0 half reflection is observed for λ_I_ = 0. E–ph coupling on the interacting dots does not change the interference conditions, it influences the correlations through the renormalization of the coupling between open dot and interacting dot and shifts of the on-site energies of IQDs. Let us examine the conductance beginning from the unperturbed IQD level. E–ph coupling breaks the SU(4) symmetry because through the coupling with phonons the intradot Coulomb interaction *U* renormalizes, whereas the interdot (or interorbital) interaction *U*’ does not change (*U* ≠ *U*’). This means that for *n* = 1 spin–charge Fano–Kondo resonance is partially destroyed, which is manifested in the changes of conductance with the increase of λ_I_.

Figure 9b compares the density of states on the IQDs and the corresponding transmission of OQDs for λ_I_ = 0.2 (*n* = 1). This picture is included as a representative example illustrating the general feature of coupling of phonons to the electrons on the interacting dots. The satellite peaks appear only in the DOS of the IQD, but their traces are not visible in transmission. This is in contrast to what is observed in coupling to an open dot. Around λ_I_ = 0.5 phonons induce a transition to the new charge state *n* = 2 (see Figure 8a) and in the transition region the most drastic changes of conductances are observed. Beyond this region conductances become constant and their values correspond to transmission of the open dots determined only by *q*, which indicates that the interacting dots are disconnected from transport. There are six states characterized by occupation *n* = 2, two with double occupancy on the single dot and a vacancy on the other dot {(0,2), (2,0)} and four states with single occupancy on each of the dots {(↑,↑), (↑,↓), (↓,↓), (↓,↓)}. For the discussed case of 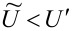 these first states are energetically lower by 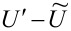 than the latter. It is well known that for strong interdot interactions (*U*’ *>> U*) electrons prefer occupy the same dot, that is, the states (2,0) and (0,2) dominate and the charge-ordered (CO) state with one of the dots being fully occupied becomes the ground state in *n* = 2 region. For *U*→∞ the dots decouple from the leads, leaving a pair of free conduction bands and a dot with degenerate charge configurations (0,2) and (2,0) [92]. It has been also predicted by the numerical renormalization group analysis performed for the system of capacitively coupled dots that the CO state occurs already for small deviations of the values of intra- and interdot interactions 
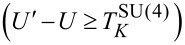
 [93]. Similarly, one can also expect the same CO ground state with (2,0) or (0,2) states for the DTQD. The SBMFA formalism we use (Equation 2 and Equation 12) with no symmetry breaking perturbation does not point directly to CO as the ground state. Instead we found a solution with two bosonic amplitudes 
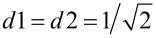
 and a vanishing rest of the amplitudes. The found SB renormailzation factor *z* (Equation 12) is equal to zero, which indicates vanishing of the Kondo scale, that is, the destruction of Kondo correlations at any finite temperature and decoupling of the interacting dots from the transport path. The obtained solution shows the destruction of the strongly correlated ground state. This suggests the conjecture, which needs however to be verified, that similarly to the isolated DTQD system, the ground state in this range is either in (0,2) or (2,0) with probability 1/2 and infinite time of transition from one degenerate state into another (*z* = 0). We have obtained clear evidence of a charge-ordered ground state in *n* = 2 domain within the SBMFA formalism by adding a small symmetry breaking term to the Hamiltonian in Equation 2. Similarly, as has been proposed in [94], we have introduced potential scattering correlated to dot occupancy 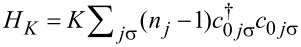. This perturbation stabilizes symmetry-broken CO state (*d*1 ≈ 1, *d*2 ≈ 0 or *d*2 ≈ 1, *d*1 ≈ 0). Hereafter this approach is called by us SB1 and is used by us only in *n* = 2 region.

**Figure 10:**
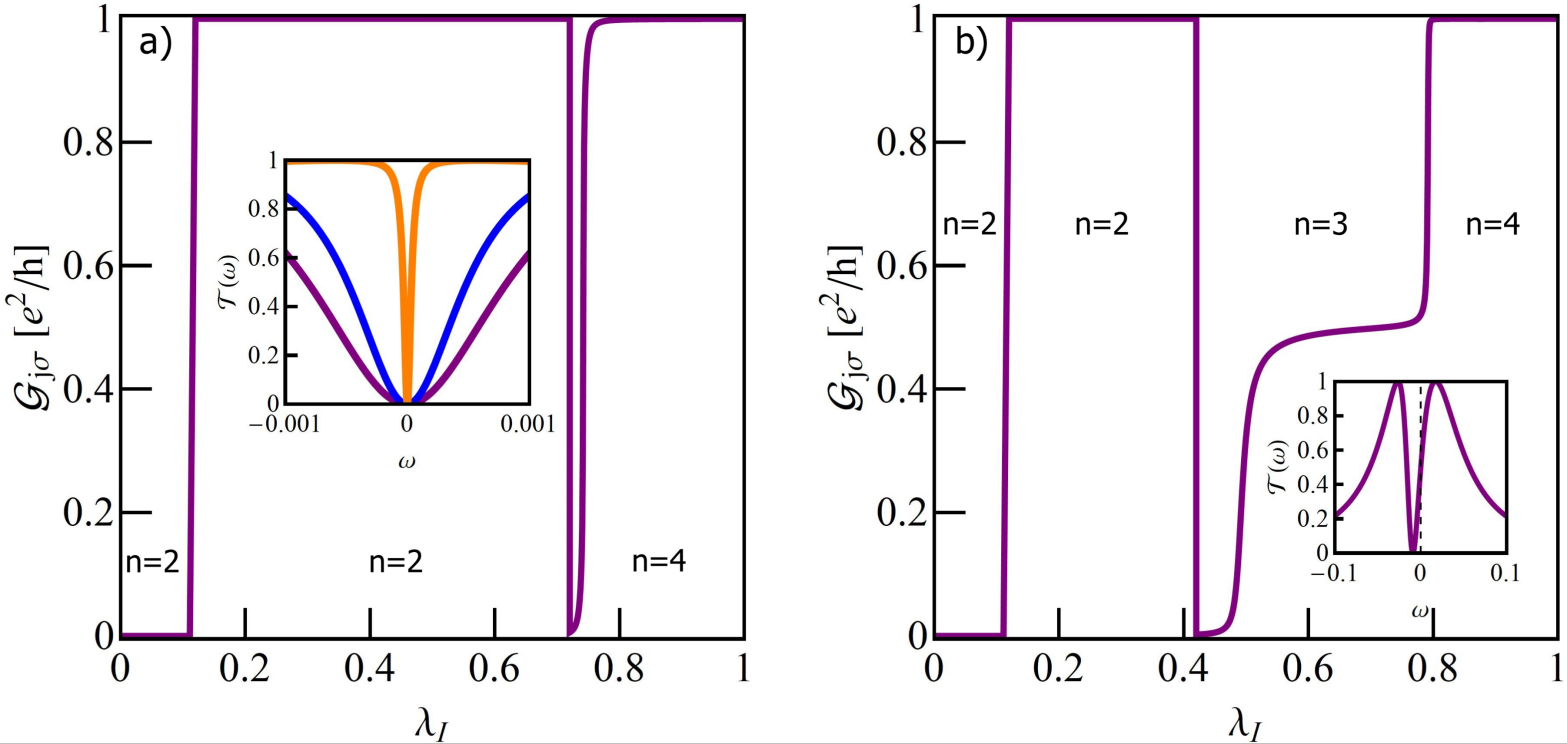
(a) Partial conductance of DTQD with a pair of phonons as a function of e-ph coupling λ_I_ with *E*_f_ = −4.5. For λ_I_ *<* 0.1 system is in the broken spin-orbital Kondo state, for λ_I_ = 0.1 charge Kondo state emerges and for λ_I_ *>* 0.1 system enters into charge ordered state (2,0) or (0,2). Around λ_I_ = 0.72 transition to the fully occupied state is observed. Inset shows transmissions for λ_I_ = 0 (purple), 0.085 (blue) and 0.095 (orange) (b) Analogous conductance dependence as in (a), but for *E*_f_ = −5.5 with additional transition to spin Kondo state for *n* = 3. Inset present transmission for λ_I_ = 0.65.

**Figure 11:**
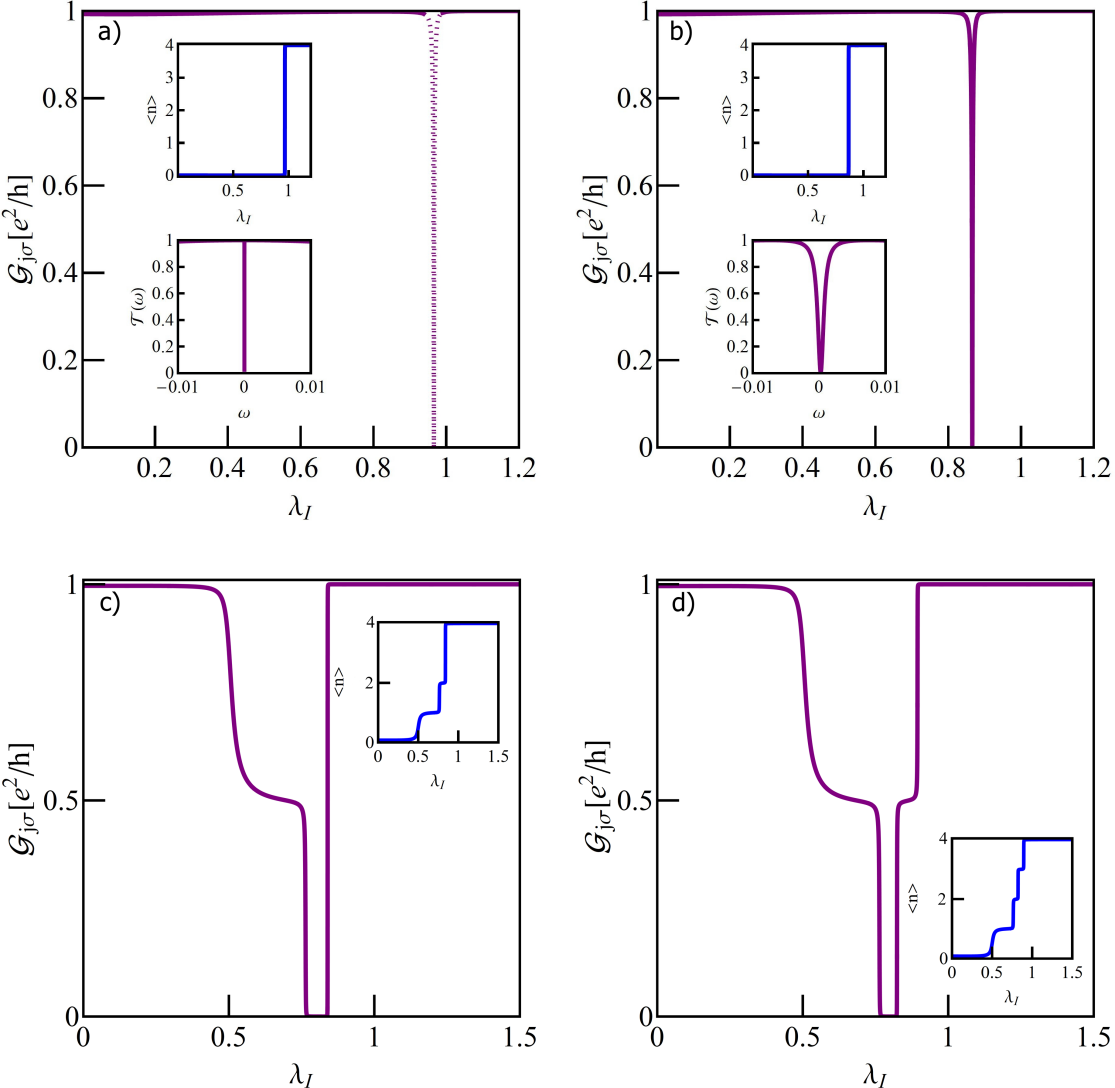
Partial conductance and occupancies (insets) of DTQD coupled to a single phonon. (a) *E*_f_ = 3 (0→4 transition with charge Kondo state for λ_I_ ≈ 0.96), (b) *E*_f_ = 1.5 (0→4 mixed valence type transition), (c) *E*_f_ = 0.5 (0→1→2→4 sequence of transitions), (d) *E*_f_ = −2 (0→1→2→3→4 sequence of transitions). Additional lower insets of (a) and (b) show corresponding transmissions close to the charge Kondo state (λ_I_ = 0.96 (a)) or in charge transition point (λ_I_ = 0.85 (b)).

Now let us look at the conductance for *q* = 0, but starting from dot energy *E*_f_ = −4.5, which already for vanishing e–ph coupling corresponds to double occupancy (*n* = 2) (Figure 10). In this case for λ_I_ = 0 SU(4) Kondo resonance is formed in the interacting dots, where all six degenerate states corresponding to *n* = 2 are engaged in spin–charge fluctuations. The resulting Kondo resonance is centered at *E*_F_ and, correspondingly, the SU(4) Fano–Kondo antiresonance has the shape of symmetric dip with zero value at the Fermi level (see inset of Figure 10a). Zero transmission corresponds to zero conductance. For increasing coupling strength, the symmetry is gradually broken and antiresonance narrows down (transmission for λ_I_ = 0.085). With the increase of λ_I_ *U*’ effectively increases with respect to *U* making the charge states {(0,2), (2,0)} lower in energy by 
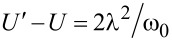
 than the spin states {(↑,↑), (↑,↓), (↓,↑), (↓,↓)}. This leads to a transition to the CO state for λ_I_ *>* λ_c_ (λ_c_ = 0.1, 
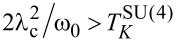
), which is well reproduced by SB1 formalism. Before this transition happens a charge Fano–Kondo state with fluctuating states {(0,2), (2,0)} is formed. The inset of Figure 10 shows densities of states at the interacting dots for λ_I_ *<* λ_c_. A considerable decrease of the widths of the resonance peaks is observed. For λ_I_ = λ_c_, where charge pseudospin is quenched, the Kondo temperature becomes extremely small. The cotunneling processes are not direct, they are mediated by higher-energy spin states (1,1), and this results in the low Kondo temperature. Up to the coupling value λ_I_ = 0.72, the DTQD remains in the degenerate charge-ordered states (0,2) or (2,0), and similarly to the previously discussed case, transport is not influenced by the interacting dots (full unitary transmission for *q* = 0). The dip of conductance occurring around λ_I_ = 0.72 reflects the transition to the state with both dots fully occupied 2→4, and for the discussed case of *q* = 0 unitary conductance is observed for *n* = 4. The transition region in the presently discussed case is much broader than presented on Figure 9 and it is related to the proximity on the energy scale of three-electron states (*n* = 3) (see Figure 8a). For slightly lower values of *E*_f_, for example, *E*_f_ = −5.5, before transition to the fully occupied state occurs, there will be a transition to *n* = 3 first (Figure 10). In the region of triple occupancy (single hole) spin–orbital SU(4) Fano–Kondo resonance is formed and half reflection is observed. The observed plateau of conductance 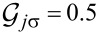 corresponds to the spin–orbital (spin–charge) SU(4) hole Fano–Kondo effect, in which participate states {|↑↓,σ⟩, |σ’,↑↓⟩}. Resonance transmission is drawn in the inset of Figure 11b. The following figures concern the case when the single-phonon mode is equally coupled to both interacting dots. In this case e–ph coupling does not break the full SU(4) symmetry.

Figure 11 shows, apart from the exemplary conductances drawn for *q* = 0 and chosen energies, the corresponding dependencies of occupations on the strength of the e–ph coupling (insets). For *E*_f_ = 3 and *E*_f_ = 1.5 the direct transition 0→4 is observed with no intermediate states. The interacting dots are disconnected from the transport when they are empty (*n* = 0) or when the two dots are fully occupied (*n* = 4). In these cases the full transmission through the OQDs occurs giving the total conductance *G*_tot_ = 2*e*^2^/*h*. However, in the transition point, the two mentioned cases are clearly different. For *E*_f_ = 1.5 all sixteen states of different occupancies are degenerate in the transition point (*e* = *p_j_*_σ_ = *d_j_* = *d*_σσ′_ = *t_j_*_σ_ = *f* = 1/4). Interacting dots in this case are in mixed valence state and real-charge fluctuations between different fillings take place. The corresponding low-energy transmissions at the open dots exhibit dips with widths only slightly reduced compared to the case of a DTQD decoupled from phonons (*z* = 1). For *E*_f_ = 3 two states degenerate at λ_I_ = λ_CK_: empty and fully occupied, and the rest of the states are energetically distant. Low-temperature physics of the DTQD in this case plays out between these two degenerate states. Effective charge fluctuations between the empty-dot system and the quadruple occupied state can be considered as isospin flips of “up” (*n* = 0) and “down” (*n* = 4) states 
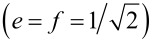
. The flips correspond to a coherent movement of four electrons into and out of the system of coupled interacting dots in the DTQD. One can suspect the occurrence of a novel charge Kondo effect for λ_CK_ = 0.9604, and the interference with direct paths leads then to the charge SU(2) Fano–Kondo antiresonance. Unfortunately, probably due to the extremely low Kondo temperature we have not succeeded numerically to find the SBMFA solutions for *E*_f_ = 3 at the very point of transition (λ_I_ = λ_CK_). Therefore, we present in the inset of Figure 11a only the transmission in vicinity of λ_CK_ (λ_I_ = 0.96). The corresponding characteristic temperature is *T*_K_ ≈ 10^−9^. Due to numerical uncertainty in the immediate vicinity of λ_CK_ the conductance and occupation lines presented on Figure 11a are drawn by dashed lines. The transition to this charge Kondo state, together with the examination of its stability under symmetry breaking perturbations, is left for more elaborated studies in the future. Figure 11 illustrates also two other cases, where between occupations 0 and 4, intermediate fillings occur for in-between values of coupling strengths. Figure 11c shows the conductance for the phonon-induced 0→1→2→4 transition and Figure 11d shows that for 0→1→2→3→4. The observed intermediate plateaus for *n* = 1, 2, and in the latter case also for *n* = 3, reflect the spin–charge (spin–orbital) SU(4) Fano–Kondo effects. The differences in the heights of plateaus for odd and even occupations are visible. In the former case the Kondo peaks at the interacting dots are shifted from the Fermi level and half reflection occurs at OQDs. In the range of *n* = 2, six two-electron states are involved in cotunneling processes and in this case Kondo resonances are centered at *E*_F_, which results in the total suppression of conductance (SU(4) Fano–Kondo antiresonance).

## Conclusion

Our analysis of the interplay of interference, strong correlations, and electron–phonon coupling is mainly addressed to molecular systems, where a strong coupling of local vibrations with electrons is expected. The discussion is also suitable for systems of suspended semiconductor-based quantum dots. It was found that transport through electron–phonon cavities with QDs embedded in a freestanding membrane is strongly affected by vibrational degrees of freedom. As model systems we have chosen single (TQD) or double (DTQD) T-shaped arrangements of quantum dots. The first system in the absence of coupling with phonons is characterized by SU(2) symmetry and the second by SU(4) symmetry. An equivalent set to a DTQD is a single TQD with orbital degeneracy. To get a more complete insight into the issues discussed we considered local phonon modes coupled either to open, noninteracting dots connected directly to the leads or vibrations coupled to the interacting dots linked to the electrodes indirectly via the open dot. Phonons interacting with electrons in the open dots form polarons and effectively renormalize coupling to the leads and shift dot site energies. This changes the interference conditions and partially suppresses correlations on the interacting dot. In consequence, modification of Fano–Kondo transmission is observed. The phononic effect gives also rise to the Franck–Condon suppression of conductance. Apart from the low-energy resonance dip, the transmission exhibits also satellite dips located at energies that coincide with multiples of the phonon energy. Due to the interactions of phonons with electrons on the interacting dot, in turn, not only the dot level position but also electron–electron interaction undergo a polaronic shift affecting the correlations. This type of coupling does not change interference conditions. Renormalizations of the electron parameters of the dots also introduce modifications of charge stability diagrams. The distance between the boundaries of adjacent regions changes with the value of the e–ph coupling parameter. In different occupation regions, apart from spin and spin–orbital Fano–Kondo resonances, also charge Fano–Kondo effects are expected. The latter occur when phonons induce the effective attraction between electrons and states with even occupancy become energetically favored. When the gate voltage is properly adjusted the energies of empty state and double or fourfold occupied states become degenerate. The low-temperature dynamics is then described only by charge fluctuations, that is, flips of charge pseudospin (charge Kondo effect). Of special interest are the effective fluctuations between degenerate empty and fully occupied states occurring in a TQD or a DTQD coupled to a single phonon mode (in the latter case phonon is equally coupled to both interacting dots (orbitals)). The double T-shaped system with two phonons separately coupled to different interacting dots is characterized by two different Coulomb interaction parameters, only one of which (intradot interaction) is renormalized by e–ph coupling. Phonon-induced transition from single to double total occupancy leads for *n* = 2 to a charge-ordered state on the interacting dots (either (2,0) or (0,2)), which results in full transmission through the open dots. For gate voltages for which the DTQD in the absence of phonons is already doubly occupied, the interaction with phonons leads with increasing coupling parameter to the evolution from SU(4) symmetry with six degenerate states to broken SU(4), where the degenerate pair (2,0) and (0,2) of charge pseudospin separate from spin states. The charge doublet lies lower on the energy scale than the spin quartet. When the phonon-induced decrease of interdot interaction parameter exceeds the energy of the SU(4) Kondo resonance, a transition to a charge-ordered state occurs, preceded at the transition point by the occurrence of a charge Kondo effect with quenched charge pseudospin.

Although T-shaped quantum dot systems discussed by us are only toy models, with the help of which we analyze the richness of emerging phenomena resulting from the interplay of three important factors, namely strong correlations, interference, and coupling with phonons, and discuss their impact on transport on a nanoscopic scale, the obtained results can be also qualitatively related to some experimental observations. Apart from many reports mentioned in the Introduction section, which demonstrate phonon-induced symmetric Kondo satellites, STM experiments presenting asymmetric satellite lines indicate the role of interference [51]. From the line widths of the central peaks or widths of satellite resonances one can infer about the Kondo temperature. In agreement with our calculations, it is observed, that *T*_K_ increases with a weakening of the e–ph coupling [95].
